# High-gain printed monopole antenna with dual-band characteristics using FSS-loading and top-hat structure

**DOI:** 10.1038/s41598-023-37186-x

**Published:** 2023-06-20

**Authors:** Patrick Danuor, Jung-Ick Moon, Young-Bae Jung

**Affiliations:** 1 Department of Electronic Engineering, Hanbat National University, Daejeon, 34158 South Korea; 2 Radio and Satellite Research Division, Electronics and Telecommunications Research Institute (ETRI), Daejeon, South Korea

**Keywords:** Electrical and electronic engineering, Optical techniques

## Abstract

In this paper, a printed monopole antenna with high-gain and dual-band characteristics for applications in wireless local area networks and the internet of things sensor networks is presented. The proposed antenna consists of a rectangular patch with multiple matching stubs surrounded to improve the impedance bandwidth of the antenna. The antenna incorporates a cross-plate structure which is seated at the base of the monopole antenna. The cross-plate consist of metallic plates aligned perpendicularly which enhances the radiations from the edges of the planar monopole to maintain uniform omnidirectional radiation patterns within the antenna’s operating band. Furthermore, a layer of frequency selective surface (FSS) unit cells and a top-hat structure is added to the antenna design. The FSS layer consist of three unit cells printed at the back side of the antenna. The top-hat structure is placed on top of the monopole antenna and comprises of three planar metallic structures arranged in a hat-like configuration. The coupling of both the FSS layer and the top-hat structure presents a large aperture to increase the directivity of the monopole antenna. Thus, the proposed antenna structure realizes a high gain without compromising the omnidirectional radiation patterns within the antenna’s operating band. A prototype of the proposed antenna is fabricated where good agreement is achieved between the measured and full-wave simulation results. The antenna achieves an impedance bandwidth |S_11_| < − 10 dB and VSWR ≤ 2 for the L and S band at 1.6–2.1 GHz and 2.4–2.85 GHz, respectively. Furthermore, a radiation efficiency of 94.2% and 89.7% is realized at 1.7 and 2.5 GHz, respectively. The proposed antenna attains a measured average gain of 5.2 dBi and 6.1 dBi at the L and S band, respectively.

## Introduction

The advancement of modern wireless systems continually places stringent requirements especially on the performance of antennas. Moreover, the rapid development of mobile communication systems and the wireless local area network (WLAN) also require antennas with high performance, omnidirectional radiation patterns and dual-band characteristics^1,2^. Microstrip antennas with compact sizes, wide bandwidth and high-gain performance features are desired for most wireless communication systems^3^.

Printed monopole antennas have received a lot of attention due to their omnidirectional radiation pattern characteristics in addition to their low cost and low profile features which makes them suitable candidates for many wireless applications including the internet of things (IoT) and WLAN^4–7^. Conventional printed monopole antennas however suffer from low gain and stable omnidirectional radiation patterns which limit their operation range^8^.

Traditional gain enhancement methods which involve an extension of the length of the monopole patch radiator or increasing the ground plane size have been proposed in a number of studies^9,10^. While these methods are effective gain enhancement techniques, they often result in antennas with large structures.

A popular gain enhancement method used nowadays involves the addition of reflective layers very close to the antenna’s radiating structure. These structures are often composed of metamaterial or periodic structures such as artificial magnetic conductors (AMC), high impedance surface (HIS), and frequency selective surfaces (FSSs) arranged in a particular geometrical manner to provide in-phase reflection to the incident signal which improves the antenna gain^11–15^. For example, an HIS structure composed of periodic square patches is implemented below the ground plane of a fork-shaped patch antenna to increase the antenna gain^11^. In Ref^12^, a wearable antenna which consists of a monopole antenna incorporated on an AMC structure for high-gain properties is presented. Also, a flexible and frequency reconfigurable monopole antenna with an FSS structure is presented for IoT applications^13^. The gain of the antenna is enhanced by placing an FSS structure beneath the antenna. In Ref^14^, a monopole directional antenna with a bioinspired elliptical leaf configuration is presented. The omnidirectional radiation pattern is converted to a directional pattern by placing a reflector near the ground plane. Again, in Ref^15^, a double split ring metasurface reflector is employed to enhance the gain of a monopole antenna. Although a significant gain enhancement is realized, large and complex structures are realized. Moreover, these gain enhancement methods result in bulky configurations since the distance between the reflective surfaces must correspond to about one-half of the wavelength.

Furthermore, the method of using superstrate layers such as partially reflecting surface (PRS) and metasurface structures to enhance the directivity of microstrip patch antennas have been presented in a number of studies^16,17^. A high-gain circularly polarized antenna, which utilizes a PRS structure placed above the microstrip patch antenna is presented in^16^. Also, a high-gain cavity resonator antenna using a metamaterial superstrate is proposed in^17^. Although a high-gain is attained with this method, large and complex antenna structures are realized. Moreover, the antenna gain enhancement is dominant only in a particular direction, thereby the omnidirectional pattern characteristics of the monopole antenna is distorted.

To attain compact high-gain monopole antennas, several antenna structures have been proposed in a number of works^18–23^. Metamaterial structures placed on or around the radiating patch of the antenna have been explored for high-gain while maintaining the compactness of the antenna^19,20^. Moreover in^21^, a circular ring-shaped monopole patch antenna on a hexagonal ground plane has been presented for high-gain characteristics. Although these structures attained high-gain with compact sizes, the omnidirectional radiation patterns are usually distorted. In Ref^22^, a compact monopole antennas with a sleeve ground plane is presented. The sleeve coupled with the ground plane form a quarter-wavelength cavity which enhances the gain. Although a stable omnidirectional radiation patterns is realized, low gain values were recorded.

A closer look at the various gain enhancement methods proposed in prior works usually involve the addition of structures that results in high-gain monopole antennas with one-sided high directive beam patterns or distorted omnidirectional radiation patterns.

In this paper, we present a novel method of realizing a printed monopole antenna with high-gain characteristics using a layer of FSS and a top-hat structure. The proposed antenna realizes high-gain with stable omnidirectional radiation patterns within the operating band of the antenna. Furthermore, the proposed monopole antenna realizes dual-band characteristics which makes it suitable for WLAN and various wireless applications including the IoT sensor networks.

## Antenna configuration

**Figure 1:**
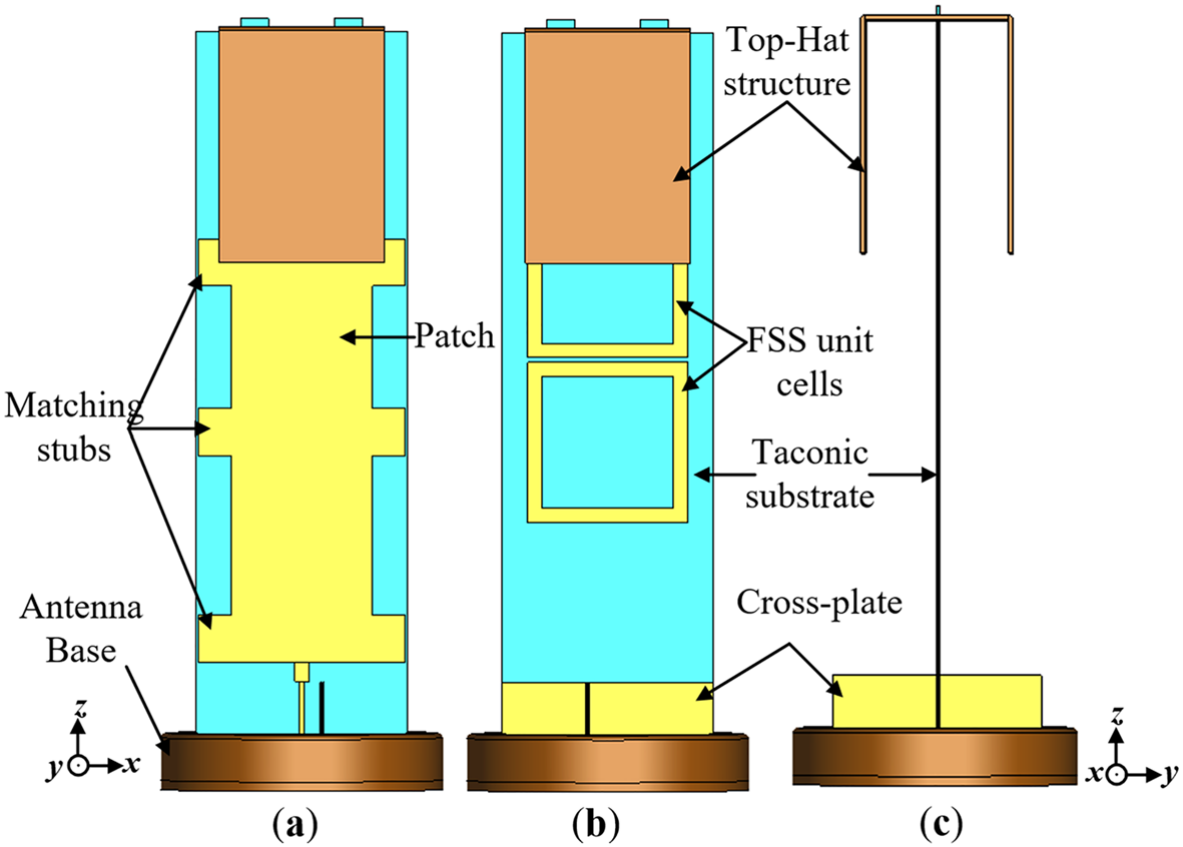
Configuration of the proposed monopole antenna (**a**) front view, (**b**) back view and (**c**) side view.

The configuration of the proposed monopole antenna is given in Fig. 1. The antenna consists of a rectangular patch radiator, a top-hat structure, three FSS unit cells and a cross-plate. The monopole radiator has a dimension of 30 mm × 130 mm while each of the FSS unit cells have a size of 34 mm × 34 mm. Both the patch and FSS are realized on a TLY-5 Taconic dielectric substrate with relative permittivity (${\varepsilon }_{r}$) of 2.2, height (*h*) of 0.508 mm and loss tangent (tan δ) of 0.0009. The top-hat structure is composed of copper metal plate arranged in a hat-like manner and is seated on the TLY-5 Taconic substrate, at the top of the monopole antenna. At the bottom part of the Taconic substrate, a metallic plate is inserted perpendicular to the printed partial ground plane at the backside of the antenna to form a cross-like printed ground plane structure.

**Figure 2:**
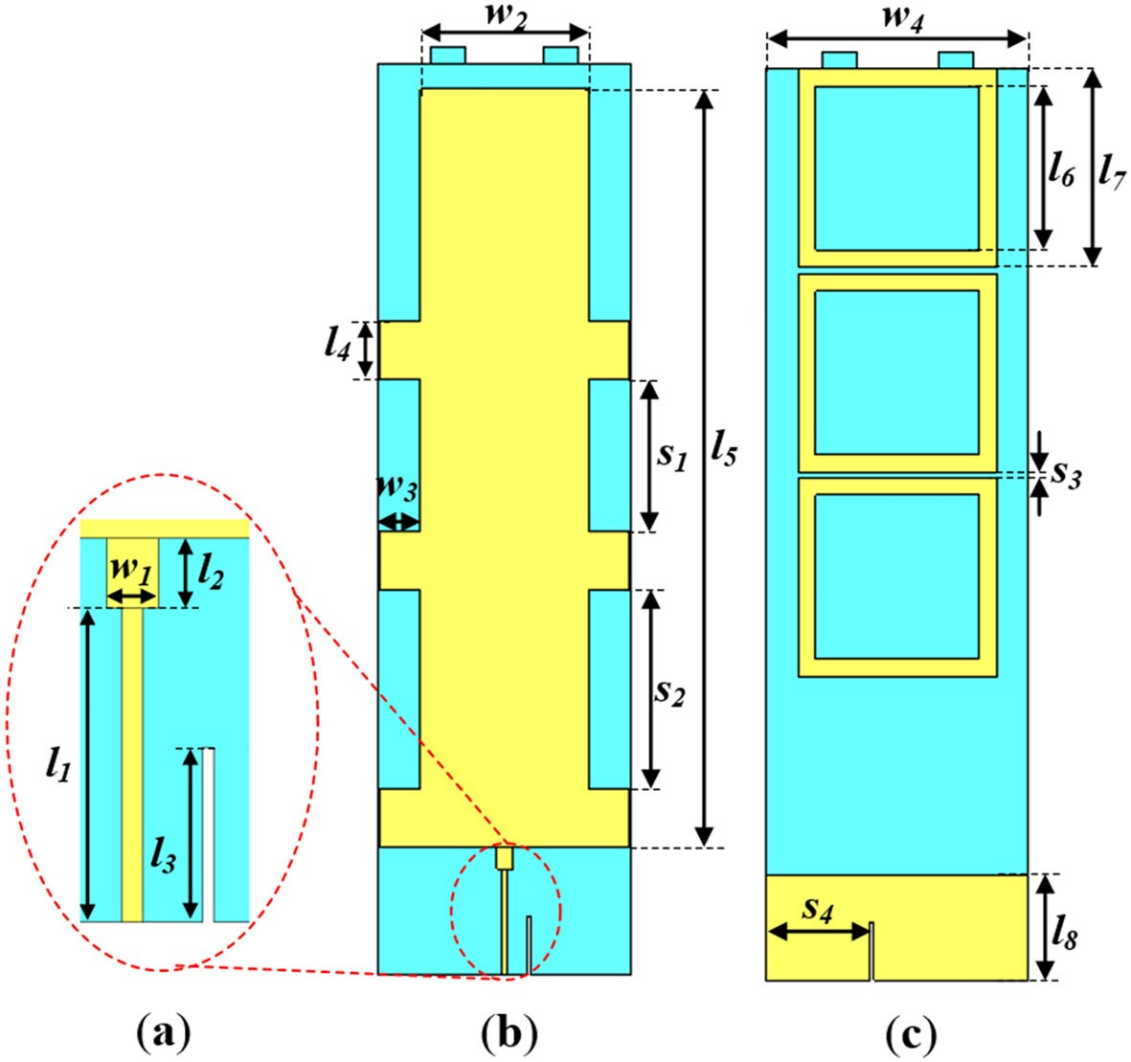
Configuration of proposed monopole antenna without top-hat and cross-plate (**a**) Exploded view of feedline with impedance transformer, (**b**) Front view and (**c**) Back view, where *l*_*1*_ = 18, *l*_*2*_ = 4, *l*_*3*_ = 10, *l*_*4*_ = 10, *l*_*5*_ = 130, *l*_*6*_ = 28, *l*_*7*_ = 34, *l*_*8*_ = 18, *w*_*1*_ = 3, *w*_*2*_ = 30, *w*_*3*_ = 7, *w*_*4*_ = 45, *s*_*1*_ = 26, *s*_*2*_ = 34, *s*_*3*_ = 1 and *s*_*4*_ = 17.92 (Unit: mm).

In the antenna design, the patch radiator is surrounded by stubs placed at optimized distances to improve the impedance bandwidth of the antenna. The rectangular patch is fed by a feedline matched to a 50 Ω-SMA connecter via an impedance transformer for measurement. The antenna is situated on a circular base with radius 30 mm, which also serves as a support to the antenna structure. The detailed dimensions of the antenna structure consisting of the patch, FSS unit cells and stubs are illustrated in Fig. 2.

## Antenna design

### Conventional monopole antenna

The conventional monopole antenna is composed of the radiating patch, feedline and the circular base. The radiating patch for the conventional monopole antenna is usually designed at a quarter-wavelength i.e., 1/4 λ_g_ (where λ_g_ corresponds to the guided wavelength). For applications in the maritime environment, the base station is usually located several meters above the antenna system embedded on the boats, thereby requiring antenna structures with tilted beam patterns. The current distributions for a dipole or monopole antenna of length 1.5λ and 0.75λ, respectively results in the tilting of the maximum beam in the upward direction. Therefore in this work, the length of the monopole patch radiator is designed at 3/4 λ_g_ (a derivative of 1/4 λ_g_) in order to generate tilted radiation pattern beams. The conventional monopole antenna exhibits an impedance bandwidth of |S_11_| < ‒10 dB at 1.35 to 1.6 GHz and 2.1 to 2.4 GHz. Moreover, a low gain below 3 dBi is realized at the lower frequency band of the antenna.

To improve the impedance matching conditions of the conventional monopole antenna, open-circuited rectangular stubs are added to the sides of the rectangular patch radiator as illustrated in Fig. 2. Each stub is designed with a length *l*_*4*_ and width *w*_*3*_.

**Figure 3:**
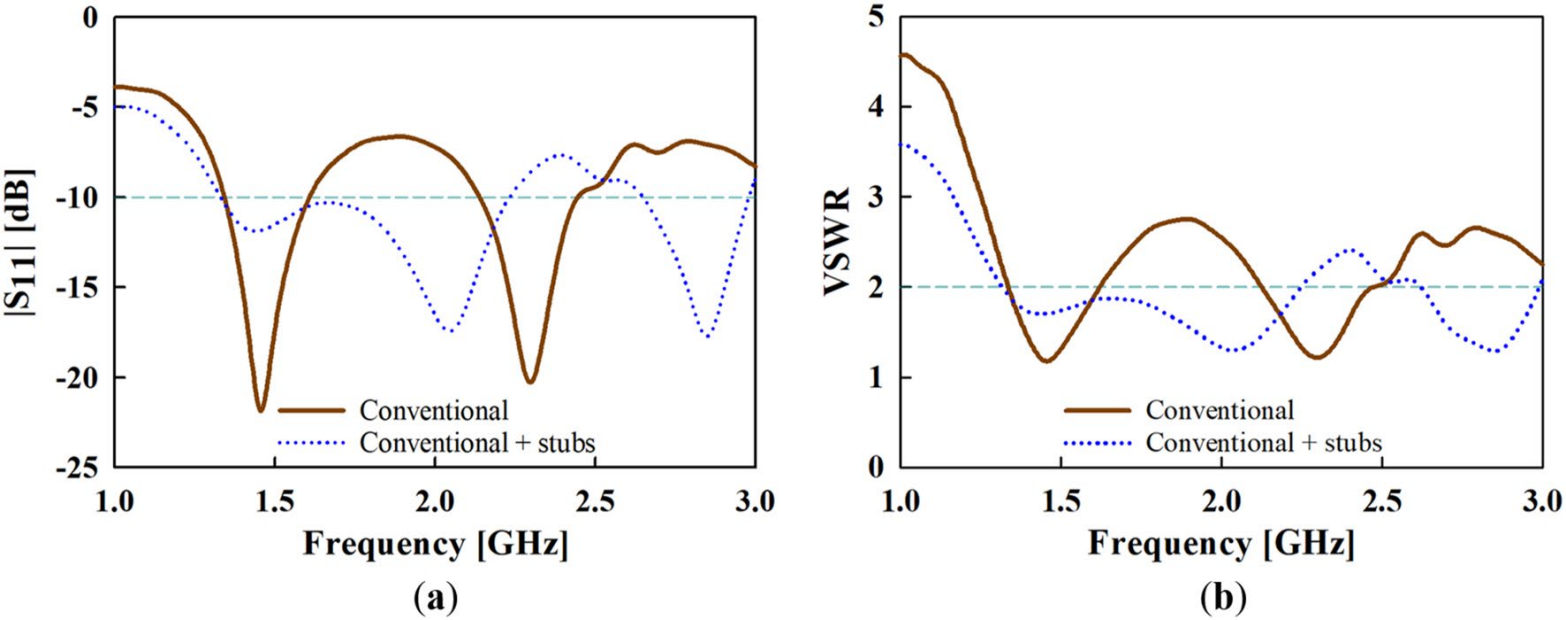
Simulated (**a**) reflection coefficient (S_11_) and (**b**) voltage wave standing ration (VSWR) results of the conventional antenna with and without the addition of stubs.

The results of the simulated reflection coefficient (S_11_) and voltage standing wave ration (VSWR) are presented and compared to the conventional one in Fig. 3a,b, respectively. An impedance bandwidth of |S_11_| < ‒10 dB and VSWR ≤ 2 at the lower frequency band of 1.32 to 2.3 GHz, and an upper frequency band of 2.6 to 2.9 GHz is achieved with the addition of stubs.

### Design of cross-plate

One of the drawbacks of the conventional printed monopole antenna is that the azimuth radiation pattern (*xoy*-plane) does not have a uniform omnidirectional pattern. This is because the radiation pattern is more directive in the *yoz*-plane compared to the *xoz*-plane. Furthermore, the omnidirectional radiation patterns of the conventional monopole antenna distorts with frequency variation within the antenna’s operating band. One way to have uniform omnidirectional radiation patterns is to reduce the width of the patch radiator. However, this approach reduces the bandwidth characteristics of the antenna.

**Figure 4:**
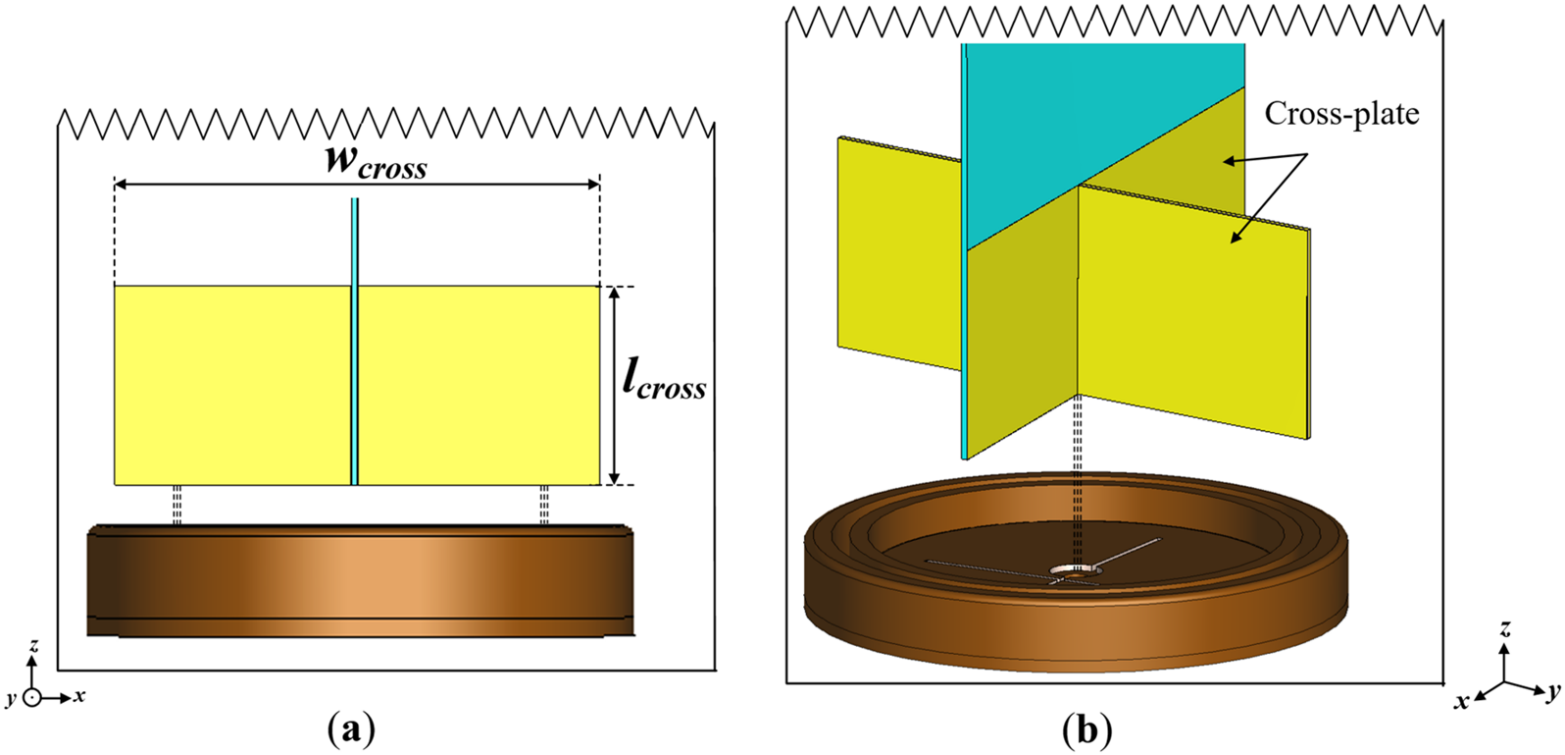
Illustration of the cross-plate incorporated into the monopole antenna design (**a**) side view and (**b**) perspective view where *l*_*cross*_ = 18 and *w*_*cross*_ = 44 (Unit: mm).

To address this challenge, a cross-plate is incorporated into the conventional monopole antenna as shown in Fig. 4. The cross-plate consist of a metallic plate inserted perpendicular to the partial ground plane which forms a cross-like structure^6^. The cross-plate structure situated on the circular-base of the antenna provides a reflective aperture to the electromagnetic waves radiated from the monopole antenna, which can prevent the azimuth radiation patterns from shrinking in a particular direction (i.e., the *yoz*-plane).

**Figure 5:**
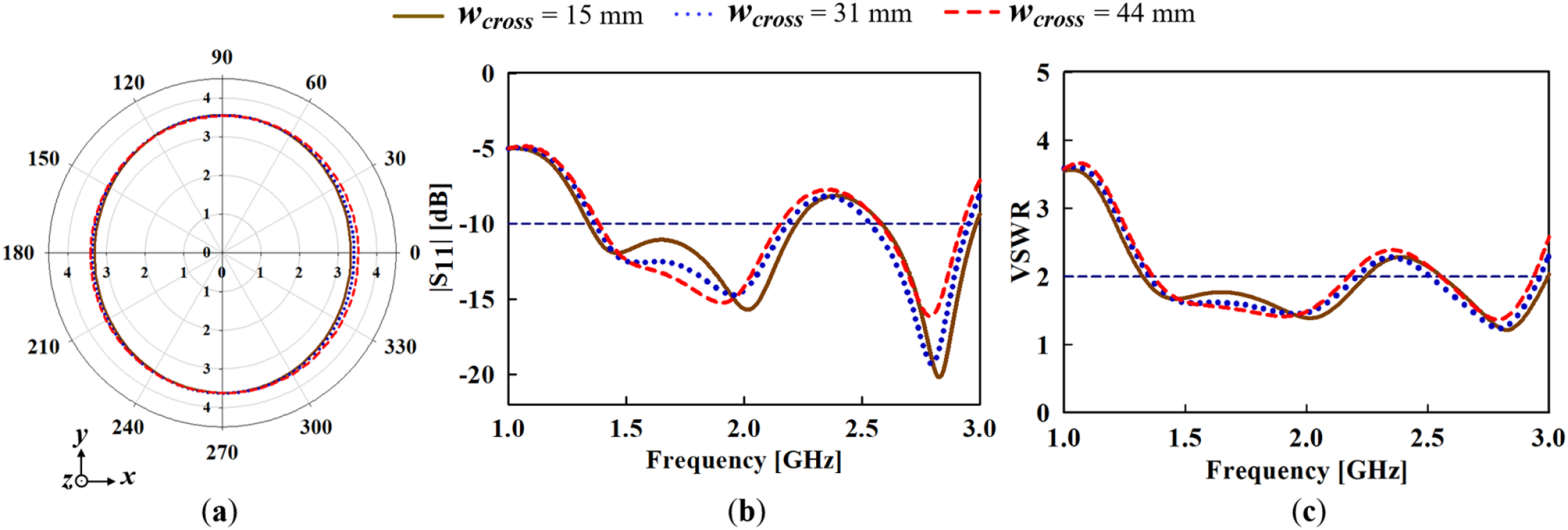
Variation of cross-plate width (*w*_*cross*_) for (**a**) azimuth radiation pattern, (**b**) simulated input reflection coefficient (S_11_) and (**c**) voltage wave standing ration (VSWR) results.

To further study the physical significance of the cross-plate, the width of the cross-plate, *w*_*cross*_ which plays a critical role in attaining a flat omnidirectional radiation pattern is varied. As shown in Fig. 5a, the azimuth radiation pattern broadens to form a more omnidirectional radiation pattern with increasing *w*_*cross*_. It can also be observed that the best azimuth radiation pattern flatness is achieved when *w*_*cross*_ = 44 mm. However, the variation of *w*_*cross*_ seems to have no significant impact on the impedance bandwidth of the monopole antenna as depicted in the S_11_ and VSWR results in Fig. 5b,c, respectively.

**Figure 6:**
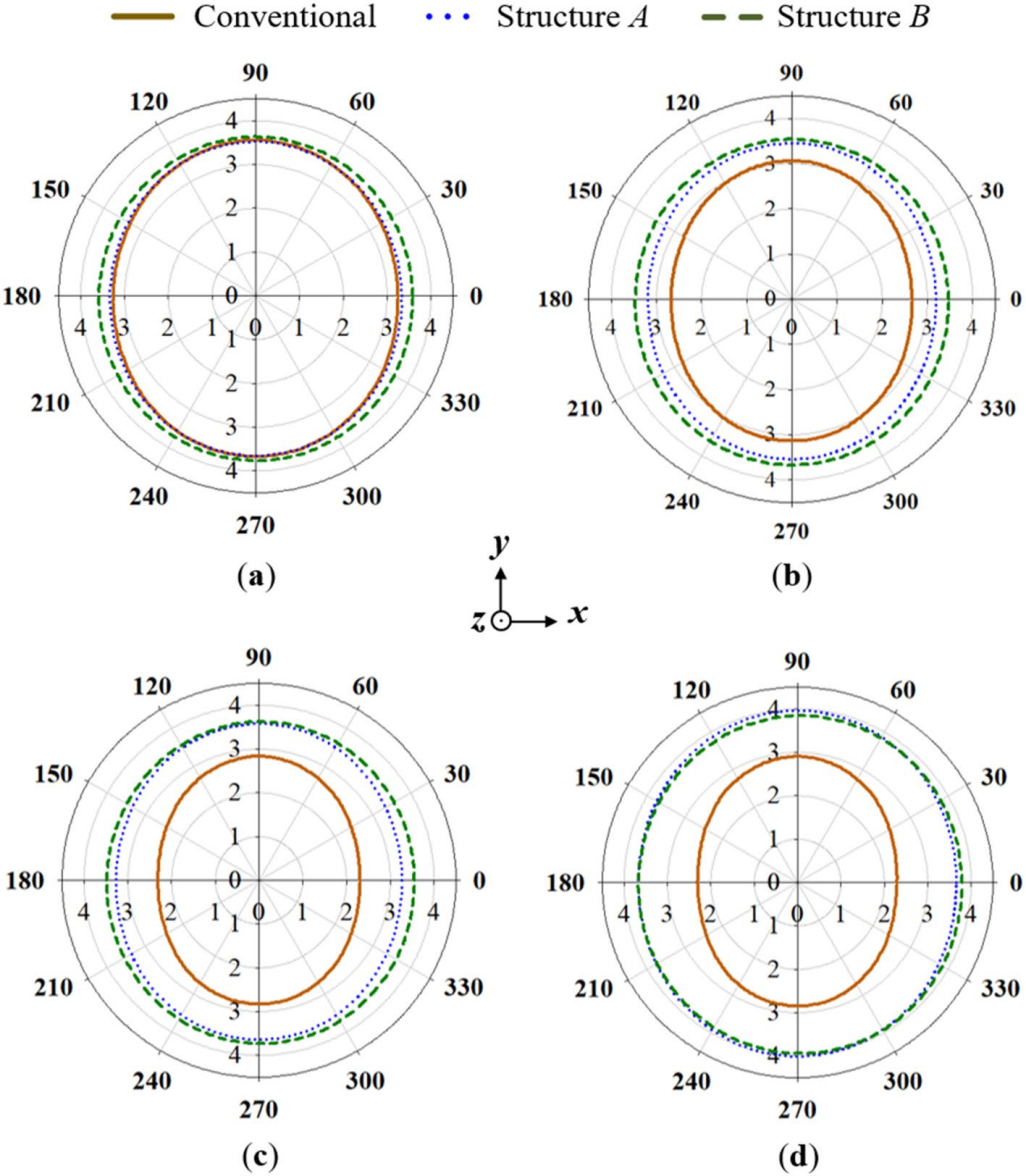
Azimuth radiation pattern results of the conventional, structure *A* and structure *B* at (**a**) 1.7 GHz, (**b**) 1.8 GHz, (**c**) 1.9 GHz and (**d**) 2 GHz.

**Figure 7:**
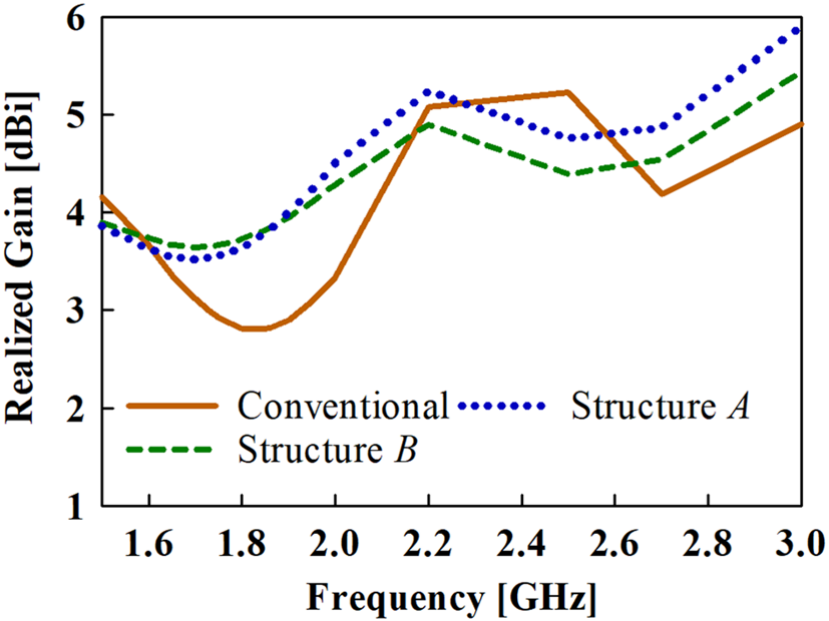
Realized gain results of the conventional antenna structures.

The azimuth radiation pattern results are given in Fig. 6. For ease of reference, the conventional monopole antenna with stubs added is labelled as Structure *A*, while the conventional monopole with both stubs and cross-plate added is labelled as Structure *B*. From the azimuth results, it can be observed that stable radiation patterns are attained when the cross-plate is added compared to the conventional monopole antenna. Furthermore, the 3D-peak gain results shown in Fig. 7 reveal a slight gain decrease in the higher frequencies when the cross-plate is added. Nevertheless, an appreciable gain increase is witnessed for the lower frequencies.

### Design of top-hat structure

**Figure 8:**
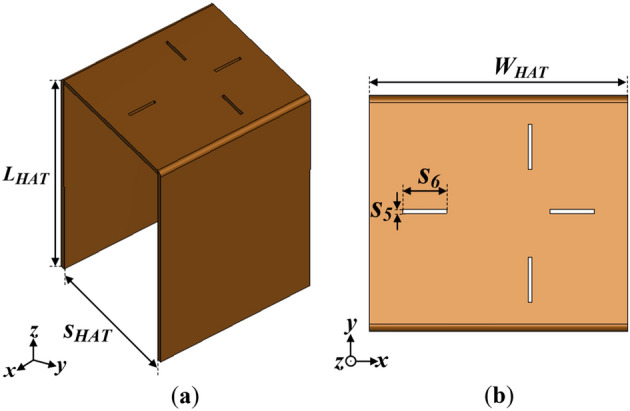
Configuration of the top-hat structure (**a**) side view and (**b**) top view where *L*_*HAT*_ = 50, and *S*_*HAT*_ = 30, *W*_*HAT*_ = 35, *s*_*5*_ = 0.57, *s*_*6*_ = 6 (Unit: mm).

To enhance the gain of the conventional monopole antenna especially with regards to the lower frequency band (i.e., the frequencies between 1.6 and 2 GHz), a top-hat structure is added at the end of the monopole antenna. The configuration of the top-hat structure is given in Fig. 8. The top-hat structure consists of three metallic sheets arranged in a hat-like configuration. The slots on the top view of the top-hat structure provides the insert points for the Taconic dielectric substrate.

The motivation behind using the metallic top-hat is to present a large radiating aperture to the monopole antenna to enhance the directivity, without increasing the overall size of the antenna. However after the incorporation of the top-hat structure, the realized gain of the monopole antenna deteriorates, although a high directivity is attained. This is mainly attributed to the poor matching conditions of the antenna after the addition of the top-hat.

In order to reap the full benefits of the top-hat structure while attaining good matching conditions of the antenna, a layer of frequency selective surface (FSS) consisting of three square-loop metallic unit cells are placed at the backside of the monopole radiating patch. FSS structures are frequency dependent surfaces composed of periodic structures that can manipulate the characteristics of the electromagnetic waves passing through it^24^. The unique frequency-shifting ability of the FSS is employed to improve the matching conditions of the antenna due to its simplicity and ease of fabrication. Moreover, the FSS structures have additional advantages of resonant properties that could be utilized to improve the impedance bandwidth characteristics of the antenna. The FSS unit cell can behave as transmissive and reflective surface based on the dimensions. Therefore, each unit cell is designed with optimized dimensions of 34 mm × 34 mm (0.3λ_g_ × 0.3λ_g_) to achieve the desired characteristics of the target band of the monopole antenna.

**Figure 9:**
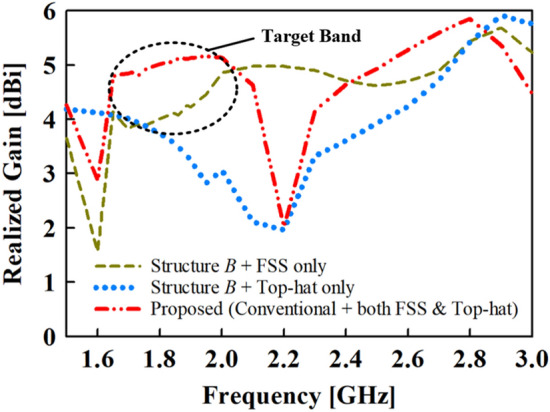
Gain results of the proposed and conventional monopole antennas.

**Figure 10:**
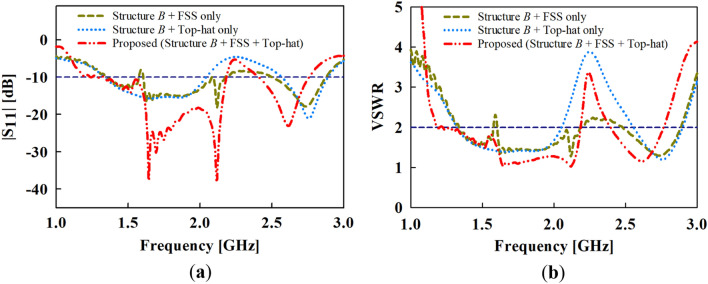
Simulated results of (**a**) reflection coefficient (S_11_) and (**b**) voltage standing wave ration (VSWR) of the proposed and conventional monopole antennas.

The top-hat structure coupled with the FSS unit cell layer results in a peak gain of about 5.2 dBi at the target band as depicted in Fig. 9. Again, it can be deduced from the results in Fig. 10 that the high-gain characteristics is achieved by both contributions of the FSS layer and the top-hat structure. The |S_11_| and VSWR results are given in Fig. 10a,b, respectively where the proposed antenna exhibits an impedance bandwidth of |S_11_| < ‒10 dB and VSWR ≤ 2 at 1.35**–**2.1 GHz and 2.4**–**2.75 GHz.

### Parametric study

**Figure 11:**
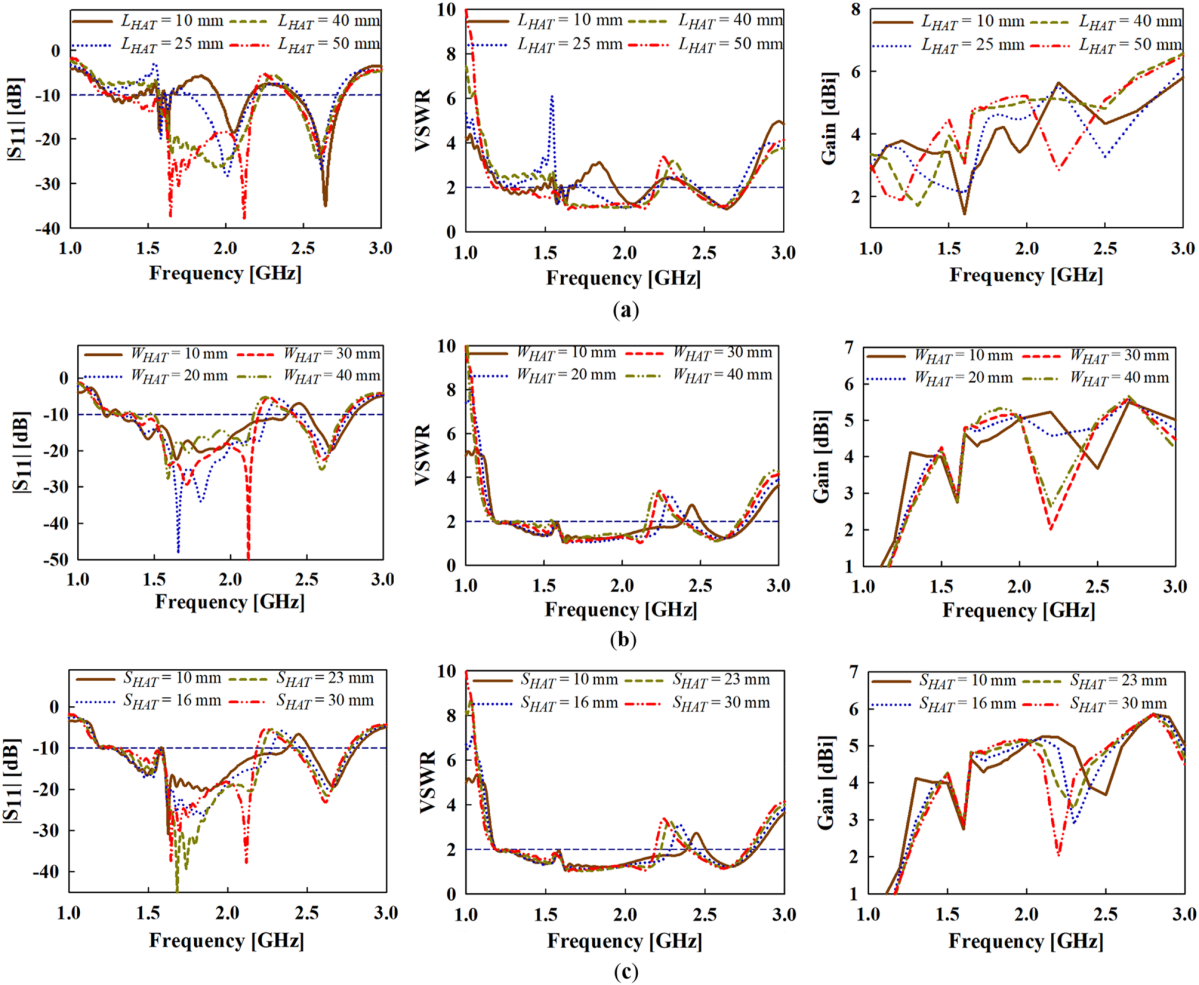
Simulated results of the |S_11_|, VSWR and Gain with varying (**a**) *L*_*HAT*_, (**b**) *W*_*HAT*_ and (**c**) *S*_*HAT*_ parameters.

To further study the effect of the key design parameters of the top-hat structure i.e., the length, width and spacing (*L*_*HAT*_, *S*_*HAT*_ and *W*_*HAT*_, respectively), a detailed parametric study in terms of the reflection coefficient (S_11_), VSWR and realized gain is given in Fig. 11a–c. With respect to the target operating band of the antenna (i.e., the lower frequencies), it can be observed that both gain and impedance bandwidth performance enhances with increasing length (*L*_*HAT*_) as shown in Fig. 11a. The best gain and bandwidth performance is achieved when *L*_*HAT*_ = 40 mm.

Moreover, increasing the width of the top-hat (*W*_*HAT*_) seems to increase the gain of the antenna at the target frequency band as shown in Fig. 11b. A trade-off between the impedance bandwidth and gain is realized in the case of *W*_*HAT*_ variation as the impedance bandwidth performance increases with decreasing *W*_*HAT*_. In this case, the best performance considering both gain and impedance is realized when *W*_*HAT*_ = 30 mm.

Furthermore, the spacing between the vertical metallic plates of top-hat (*S*_*HAT*_) is varied to determine its impact on the gain and impedance bandwidth performance as shown in Fig. 11c. Again, a similar observation can be made for *S*_*HAT*_ as in the case of *W*_*HAT*_ that, a trade-off occurs between the gain and impedance bandwidth performance. From the results in Fig. 11c, the best gain and bandwidth performance is achieved when *S*_*HAT*_ = 30 mm.

## Measurement results and discussion

**Figure 12:**
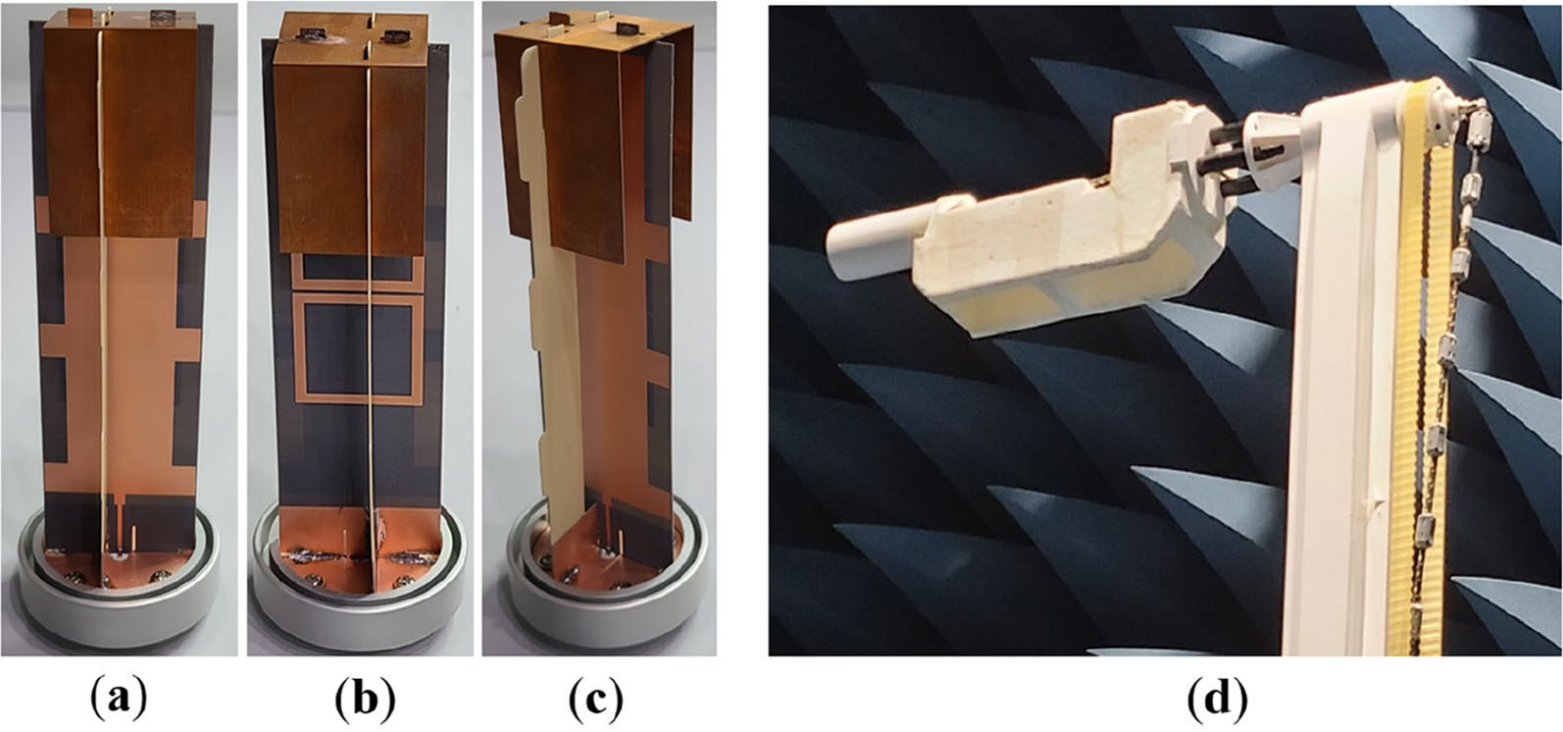
Photograph of the fabricated proposed high-gain monopole antenna (**a**) Front view, (**b**) Back view, (**c**) Side view and (**d**) Far-field measurement set-up.

To verify the proposed high-gain printed monopole antenna, a prototype is fabricated as shown in Fig. 12. A supporting structure, made up of FR-4 substrate is incorporated into the fabricated antenna to provide support to the top-hat structure as depicted in Fig. 12. The supporting structure has a negligible impact on the performance of the monopole antenna.

**Figure 13:**
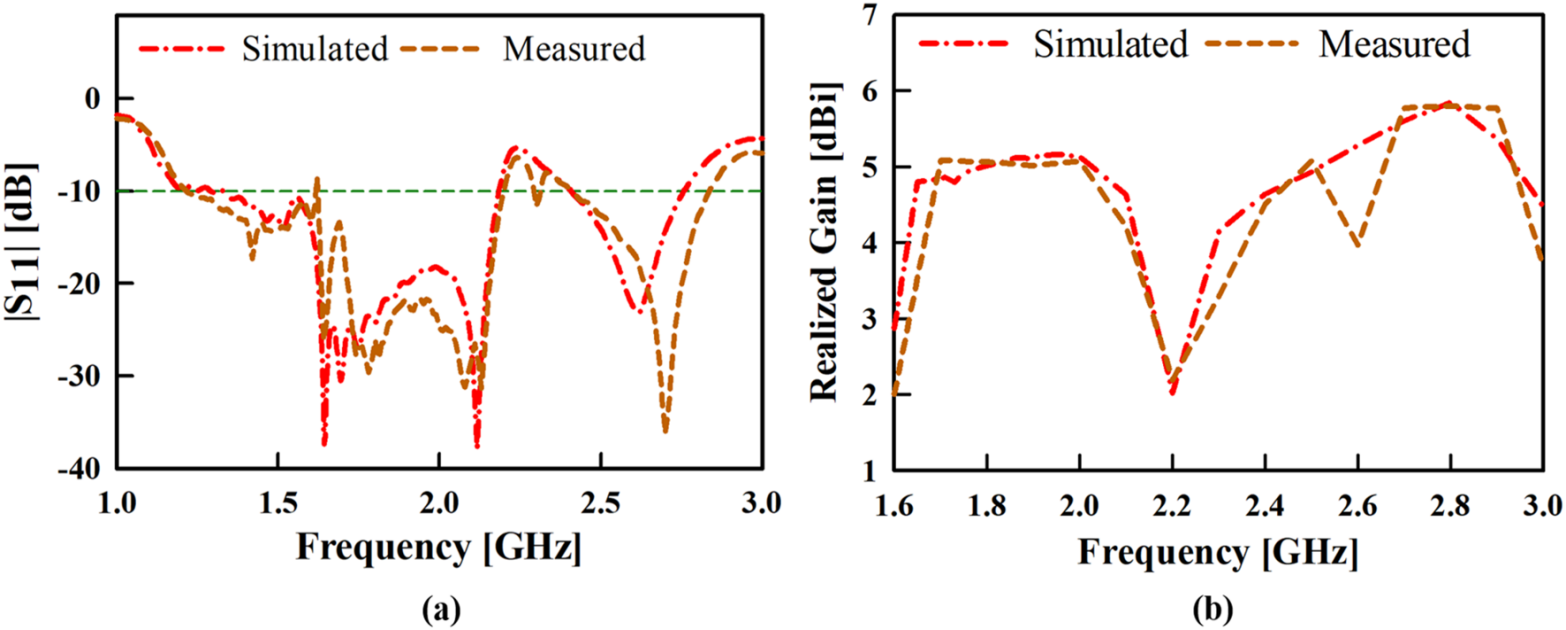
Simulated and measured results of the proposed antenna (**a**) input reflection coefficient amplitude and (**b**) realized gain.

The simulated and measured |S_11_| results of the proposed antenna are given and compared in Fig. 13a which shows good agreement in the impedance bandwidth, |S_11_|< **–**10 dB at 1.6 to 2.1 GHz (BW = 27%) and at 2.4 to 2.85 GHz (BW = 17.14%).

In addition, the simulated and measured gain results of the proposed antenna are compared in Fig. 13b. The proposed antenna attained a measured gain of about 5.2 dBi throughout the lower frequency band and a measured peak gain of about 6.1 dBi at 2.8 GHz.

**Figure 14:**
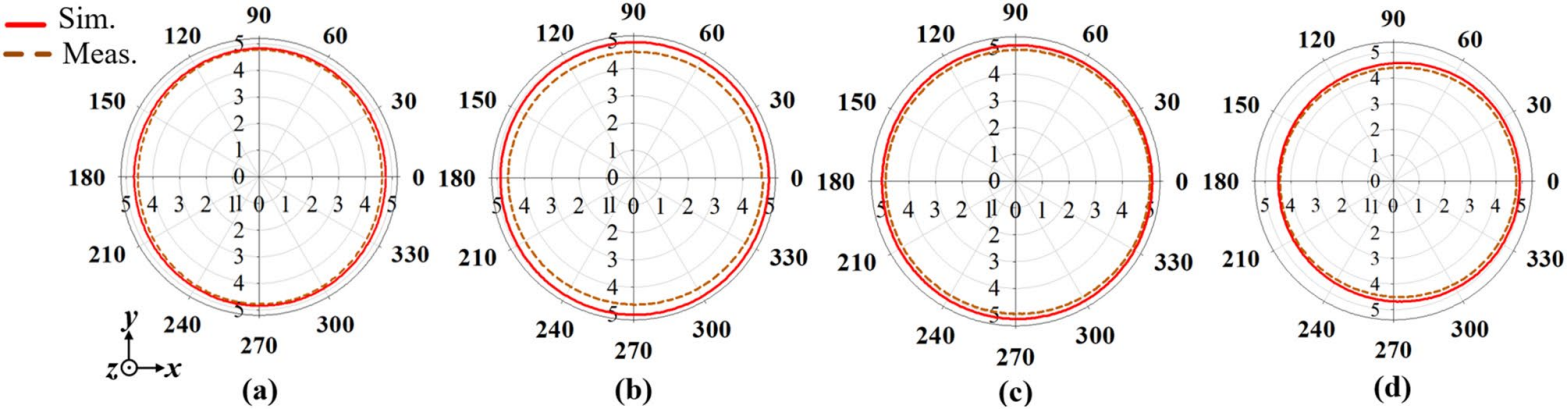
Simulated and measured azimuth patterns of the proposed antenna at (**a**) 1.7, (**b**) 1.8, (**c**) 1.9 and (**d**) 2.5 GHz.

**Figure 15:**
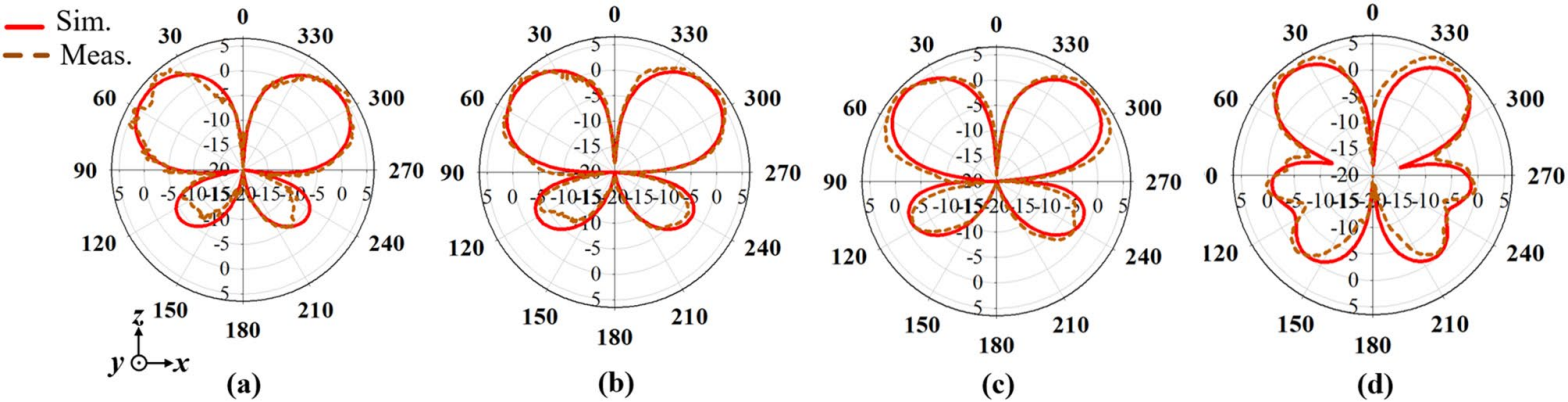
Simulated and measured elevation (*xoz*-plane) patterns of the proposed antenna at (**a**) 1.7, (**b**) 1.8, (**c**) 1.9 and (**d**) 2.5 GHz.

**Figure 16:**
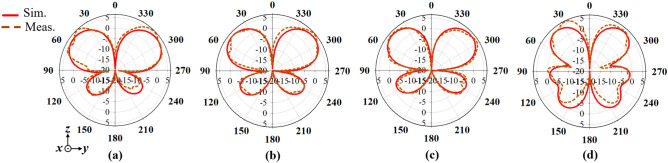
Simulated and measured elevation (*yoz*-plane) patterns of the proposed antenna at (**a**) 1.7, (**b**) 1.8, (**c**) 1.9 and (**d**) 2.5 GHz.

Moreover, Figs. 14, 15 and 16 presents the simulated and measured azimuth (*xoy-plane)*, elevation (*xoz-plane)* and elevation (*yoz-plane)* radiation patterns, respectively at 1.7, 1.8, 1.9 and 2 GHz. It can be observed from the azimuth radiation patterns that the proposed antenna maintains a stable and uniform omnidirectional radiation patterns within the antenna’s operating band. Furthermore, a 3 dB-beamwidth of 46.4°, 49.0°, 41.7° and 36.0° at 1.7, 1.8, 1.9 and 2.5 GHz, respectively is observed for the elevation (*xoz*-plane) shown in Figs. 15, and in 16, a 3 dB-beamwidth of 46.6°, 44.2°, 42.1° and 35.6° is observed at 1.7, 1.8, 1.9 and 2.5 GHz, respectively for the elevation (*yoz*-plane) radiation patterns of the proposed antenna. Both the measured and simulated patterns agree well which shows that the proposed antenna attains stable and uniform omnidirectional radiation patterns within its operating band.

The performance of the proposed high-gain monopole antenna is compared with existing monopole antenna structures in Table 1. The proposed antenna offers a comparatively higher gain than the reference antennas, and similar gain performance to Ref^11^, however distorted omnidirectional patterns are observed for Ref^11^. In addition, the proposed antenna realizes a good impedance bandwidth performance compared to the reference antennas. Also, the proposed antenna achieves a good radiation efficiency comparatively. The high-gain characteristics with the added advantages of stable omnidirectional radiation patterns highlights the proposed antenna as suitable for application in wireless communications and WLAN sensor networks.

**Table 1:** Comparison between the proposed antenna and existing antenna designs.

References	Impedance BW (GHz)	Peak gain (dBi)	3-dB gain bandwidth (%)	Antenna size (W × L)	Radiation pattern	Applications	Antenna efficiency (%)
^4^	2.15–2.65	2.39	–	37 × 60 mm^2^	Distorted omnidirectional	WLAN/Wi-Fi/WiMAX	–
	4.85–5.92	4.51		0.27λ_0_ × 0.48λ_0_			
^5^	2.38–2.75	4.4	2.6	42 × 48.5 mm^2^	Good omnidirectional	WLAN	–
	4.05–6.38	7.5	15.25	0.36λ_0_ × 0.41λ_0_			
^7^	1.81–3.83	2.5	–	63 × 75 mm^2^	Distorted omnidirectional	WLAN/WiMAX	90
				0.60λ_0_ × 0.71λ_0_			
^11^	4.30–5.90	6.12	–	102 × 68 mm^2^	Distorted omnidirectional	Wearable wireless devices	–
				1.73λ_0_ × 1.16λ_0_			
^17^	1.82–1.98	4.3	–	86.5 × 86.5 mm^2^	Distorted omnidirectional	Wearable wireless devices	98
				0.55λ_0_ × 0.55λ_0_			
^18^	2.3–4	3.2	–	40 × 45 mm^2^	Good omnidirectional	WLAN/WiMAX	76.5
	5–6.6	2.34		0.42λ_0_ × 0.47λ_0_			
^21^	2.38–2.54	3.6	10.1	29 × 100 mm^2^	Good omnidirectional	WLAN	–
				0.23λ_0_ × 0.82λ_0_			
^22^	1.81–2.08	1.92	–	30 × 55 mm^2^	Distorted omnidirectional	Mobile com. and WLAN	–
	2.36–2.70	2.12		0.19λ_0_ × 0.35λ_0_			
This work	1.6–2.1	5.2	27	45 × 156 mm^2^	Good omnidirectional	IoT and WLAN	93.6
	2.4–2.85	6.1	17.14	0.27λ_0_ × 0.94λ_0_			

## Conclusion

In this paper, a novel method of enhancing the gain of a printed monopole antenna while maintaining the omnidirectional radiation pattern characteristics throughout its operating frequency band has been presented. The presented antenna structure is composed of a rectangular patch surrounded by multiple stubs for impedance bandwidth enhancement. To attain good omnidirectional patterns within the antenna’s operating band, a cross-plate structure is incorporated into the base of the antenna to enhance the edge radiations of the monopole antenna thereby preventing shrinking of the omnidirectional radiation patterns. Furthermore, a top-hat and a layer of FSS unit cells, printed at the backside of the antenna is incorporated, which increases the effective aperture of the antenna and enhances the antenna gain. The proposed antenna realizes dual-band characteristics in the L and S band at 1.6 to 2.1 GHz, and 2.4 to 2.85 GHz, respectively. A peak gain of 5.2 dBi and 6.1 dBi is realized for the L and S band, with an average radiation intensity of 94% and 89%, respectively. The proposed monopole antenna has a high-gain with the added benefits of stable omnidirectional radiation patterns which makes it suitable for applications in wireless communications and IoT sensor newtorks, and satisfies the stringent requirement of omnidirectional patterns needed for WLAN applications.

## Data Availability

The datasets used and/or analysed during the current study are available from the corresponding author on reasonable request.
